# Supplementary figures and images for: Acid stress signals are integrated into the σB-dependent general stress response pathway via the stressosome in the food-borne pathogen Listeria monocytogenes
Source: PLoS Pathog. 2022 Mar 11;18(3):e1010213. doi: 10.1371/journal.ppat.1010213 (PMC8942246; doi:10.1371/journal.ppat.1010213)

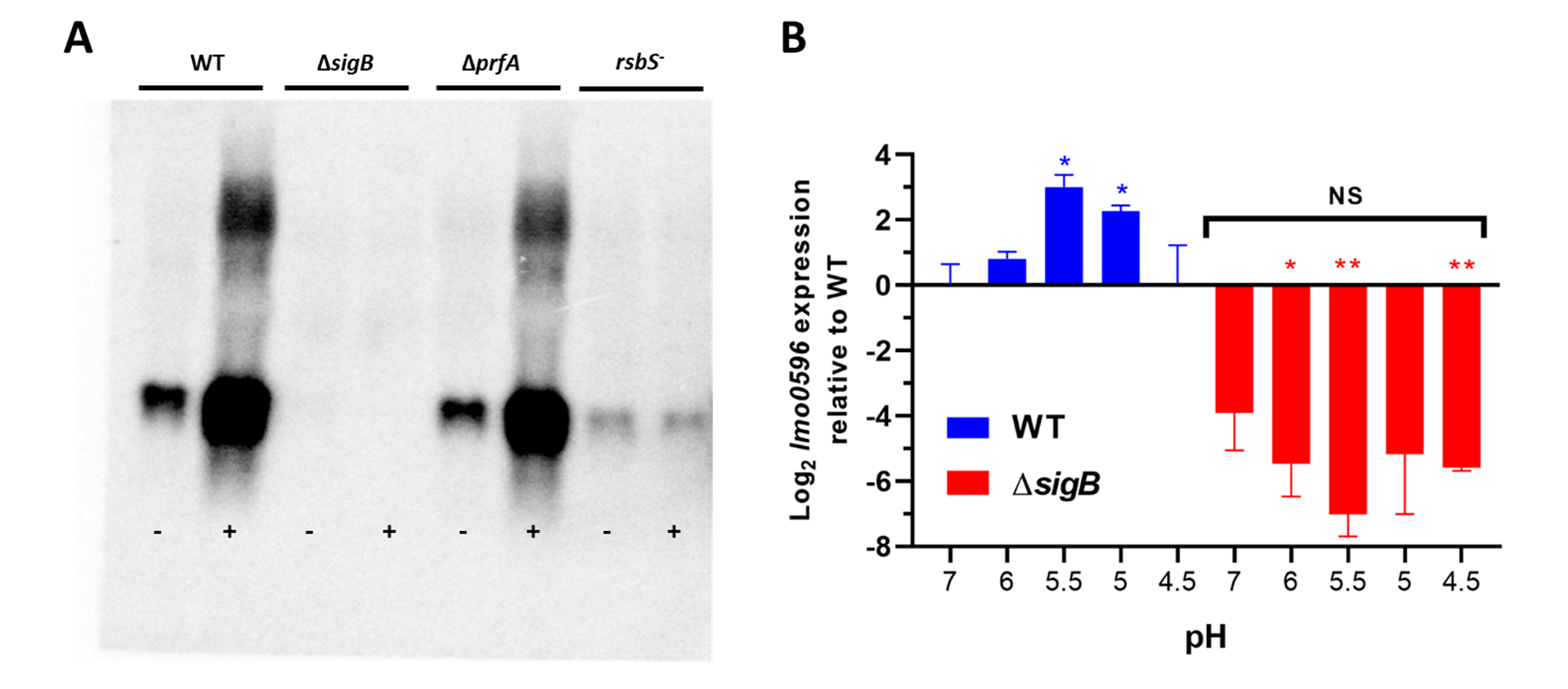

Supplement: S1 Fig — Cultures of wild type, ΔsigB, ΔprfA and rsbS- (formerly C12:C14) strains were grown to late-log phase (OD600 = 0.8) and untreated (-) and treated (+) samples in pH 5.0 or a range of pH (7.0 to 4.5) for 15 min. Total RNA was extracted as described in material and methods. rsbS- mutant consists on a transposon strain which also carries a frameshift in rsbS resulting in the premature stop codon and a polar effect on RsbT translation (57). (A) Northern blots probed for lmo0596. (B) RT-qPCR results for lmo0596 expression in wild type and ΔsigB strains in BHI adjusted to a pH range (7, 6, 5.5, 5 and 4.5). Gene expression obtained by RT-qPCR is expressed as Log2 relative gene expression. Statistical analysis was performed using a paired student t test relative to the untreated wild type after 15 min (NS = not significant; *, P value of <0.05; **, P value of <0.01; ***, P value of <0.001). (TIF) [file ppat.1010213.s001.tif]

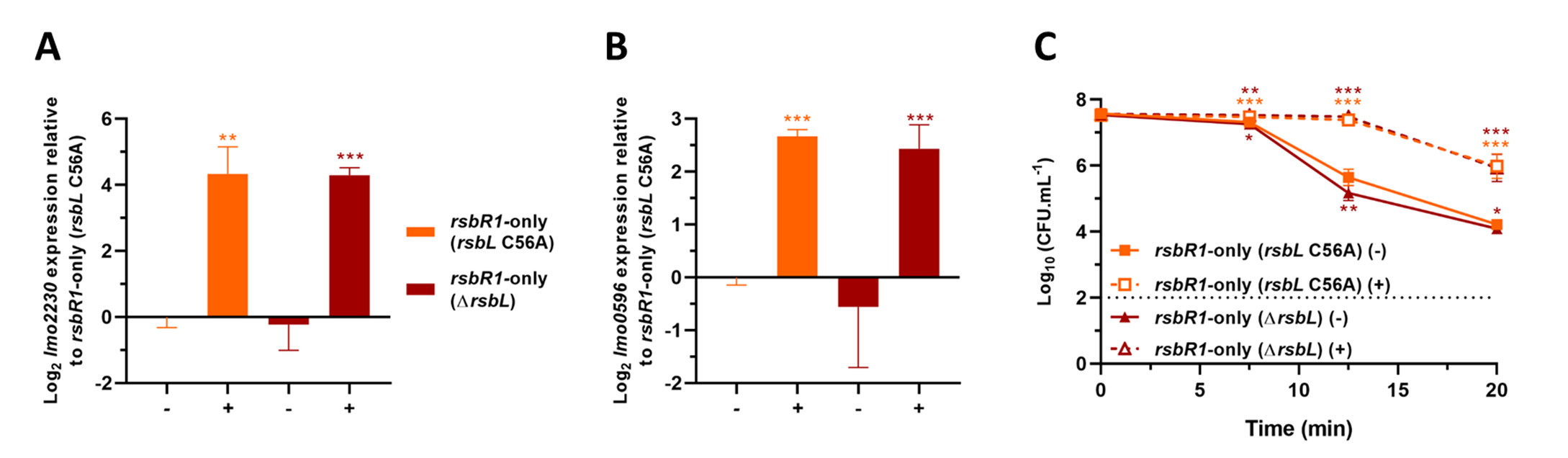

Supplement: S2 Fig — Expression of σB-dependent genes (A) lmo2230 and (B) lmo0596, obtained from mid-log phase cultures of rsbR1-only (rsbL C56A) and rsbR1-only (ΔrsbL) strains untreated (-) and treated (+) for 15 min in pH 5.0 at 37°C. (C) Acid challenge of mid-log phase cultures treated in pH 5.0 for 15 min at 37°C, as previously described in Fig 2D. Survival data is expressed as Log10 (CFU.mL-1). The rsbR1-only (rsbL C56A) genotype ΔrsbR2 ΔrsbR3 ΔrsbR4 rsbL (C56A) and the genotype of rsbR1-only (ΔrsbL) consists on ΔrsbR2 ΔrsbR3 ΔrsbR4 ΔrsbL. Statistical analysis was performed using a paired student t test relative to the untreated rsbR1-only (rsbL C56A) after 15 min, for RT-qPCR data and untreated wild type at time 0 min, for acid challenge data. (*, P value of <0.05; **, P value of <0.01; ***, P value of <0.001). (TIF) [file ppat.1010213.s002.tif]

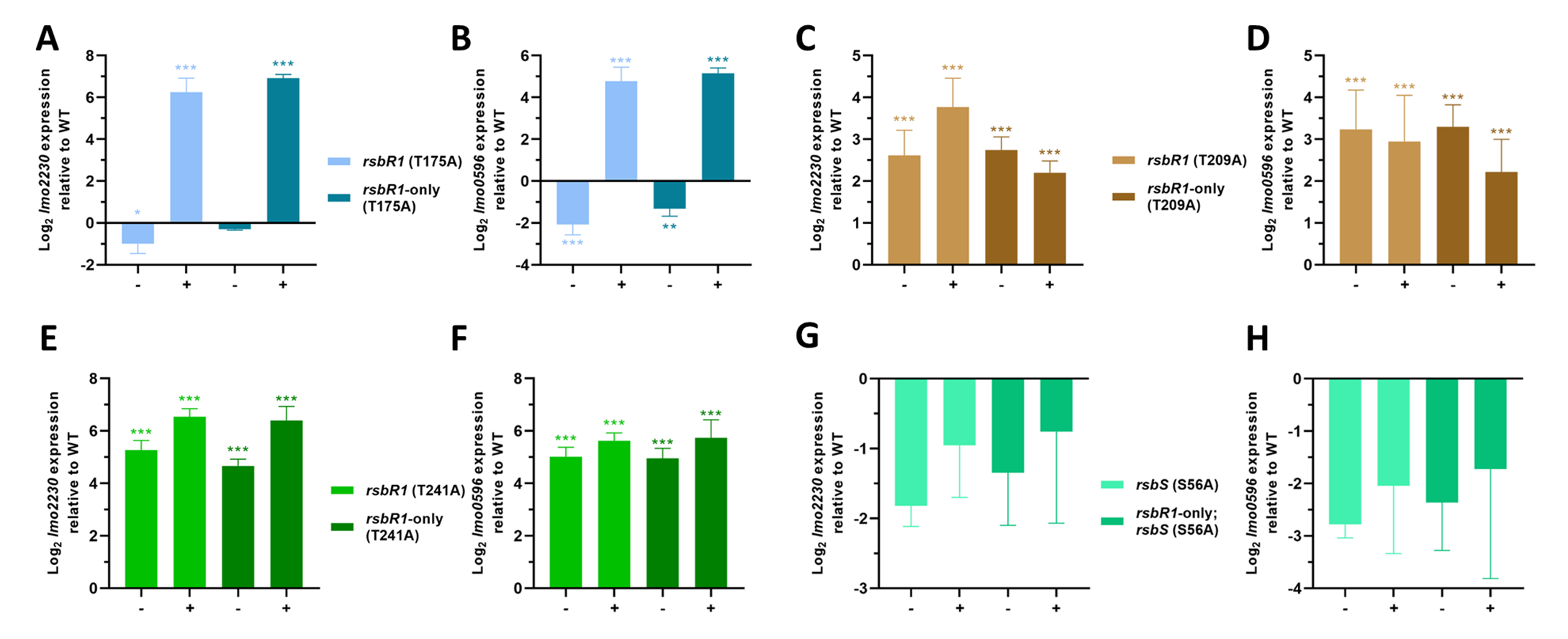

Supplement: S3 Fig — Expression of σB-dependent genes (A, C, E, G) lmo2230 and (B, D, F, H) lmo0596, obtained from mid-log phase cultures of rsbR1 (T175A), rsbR1 (T209A), rsbR1 (T241A), rsbS (S56A) and their respective rsbR1-only backgrounds. These strains were untreated (-) and treated (+) for 15 min in pH 5.0 at 37°C. The rsbR1-only backgrounds consist on the following genotype ΔrsbR2; ΔrsbR3; ΔrsbR4; rsbL (C56A). Statistical analysis was performed using a paired student t test relative to the untreated wild type strain after 15 min (*, P value of <0.05; **, P value of <0.01; ***, P value of <0.001). (TIF) [file ppat.1010213.s003.tif]

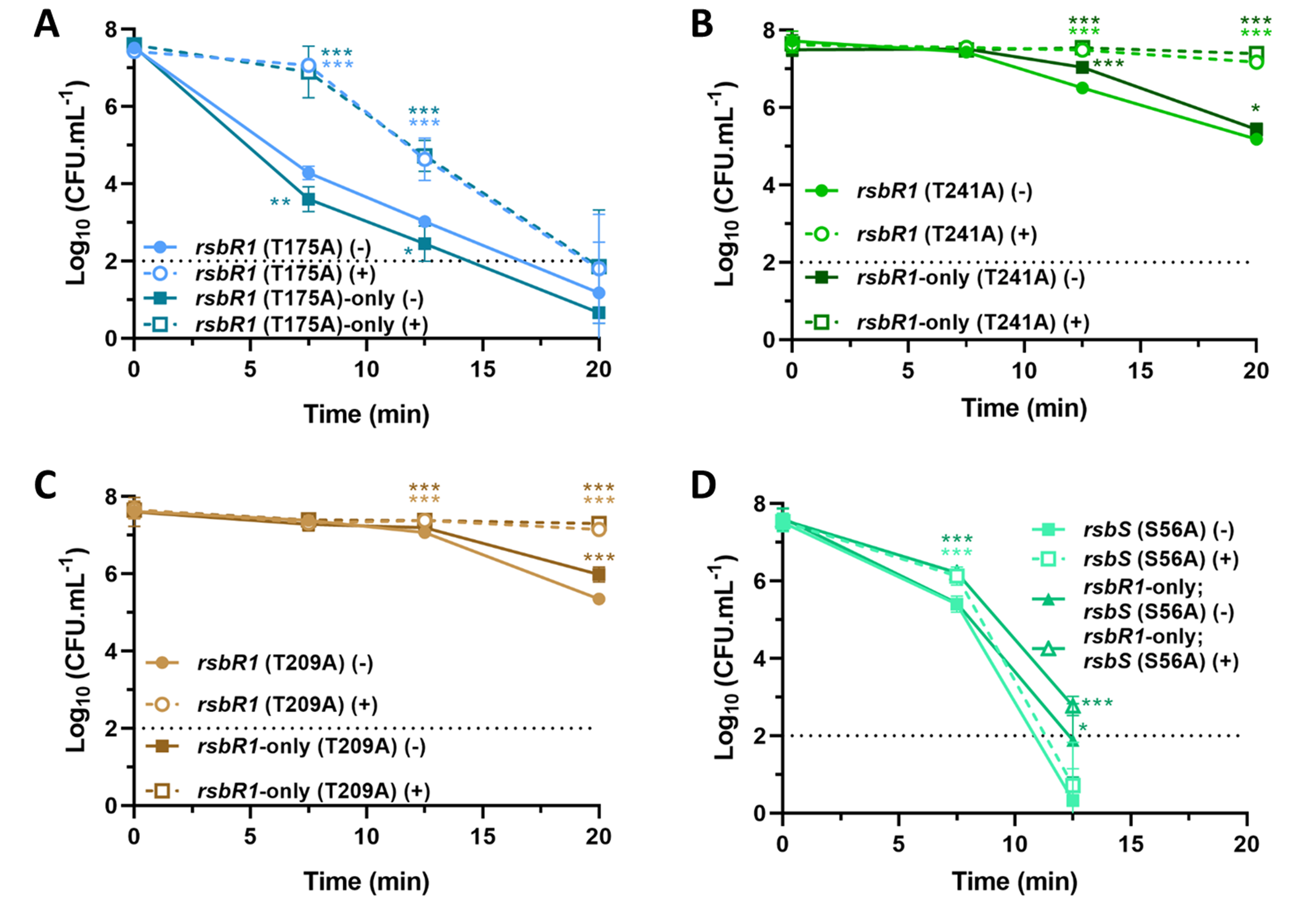

Supplement: S4 Fig — Acid challenge of mid-log phase cultures of (A) rsbR1 (T175A), (B) rsbR1 (T209A), (C) rsbR1 (T241A), (D) rsbS (S56A) and their respective rsbR1-only backgrounds, treated in BHI acidified to pH 5.0 for 15 min at 37°C. Survival data is expressed as Log10 (CFU.mL-1). The rsbR1-only backgrounds consist in ΔrsbR2; ΔrsbR3; ΔrsbR4; rsbL (C56A). The dashed line represents the detection threshold. Samples were taken at 0, 7.5, 12.5 and 20 min. Survival data is expressed as Log10 (CFU.mL-1). Statistical analysis was performed using a paired student t test relative to the respective untreated parental strain after at time 0 min (*, P value of <0.05; **, P value of <0.01; ***, P value of <0.001). (TIF) [file ppat.1010213.s004.tif]

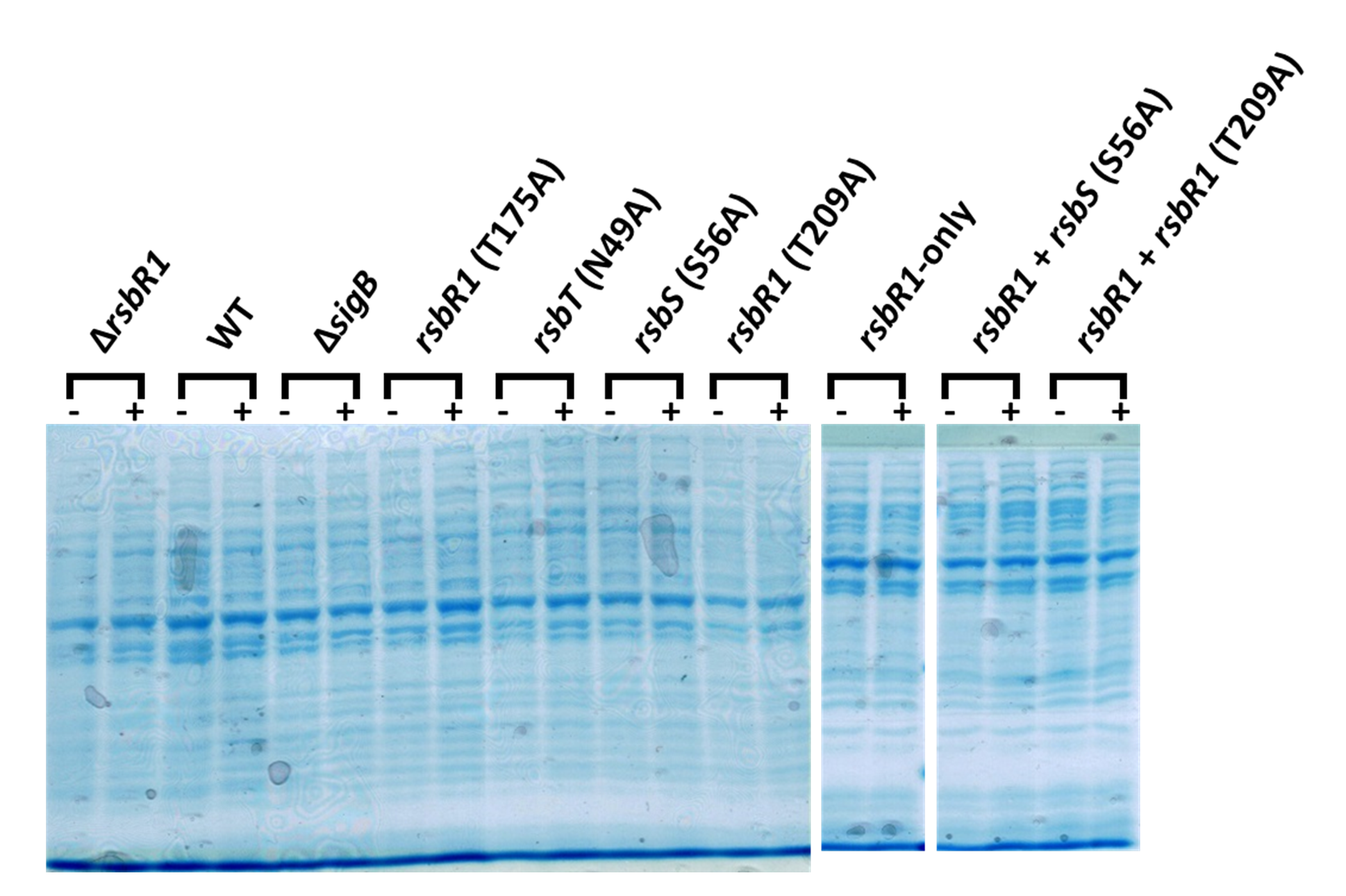

Supplement: S5 Fig — SDS-PAGE gels of normalized total protein extracts used for the Phos-tag and western-blots made in this study. Total protein extractions were obtain from mid-log phase cultures untreated (-) and treated (+) in pH 5.0 for 15 min at 37°C. Each protein sample was obtained from ~7 x 10^7^ cells. Total protein was separated in 12% acrylamide/bis-acrylamide gels and subsequently stained with Coomassie dye. (TIF) [file ppat.1010213.s005.tif]

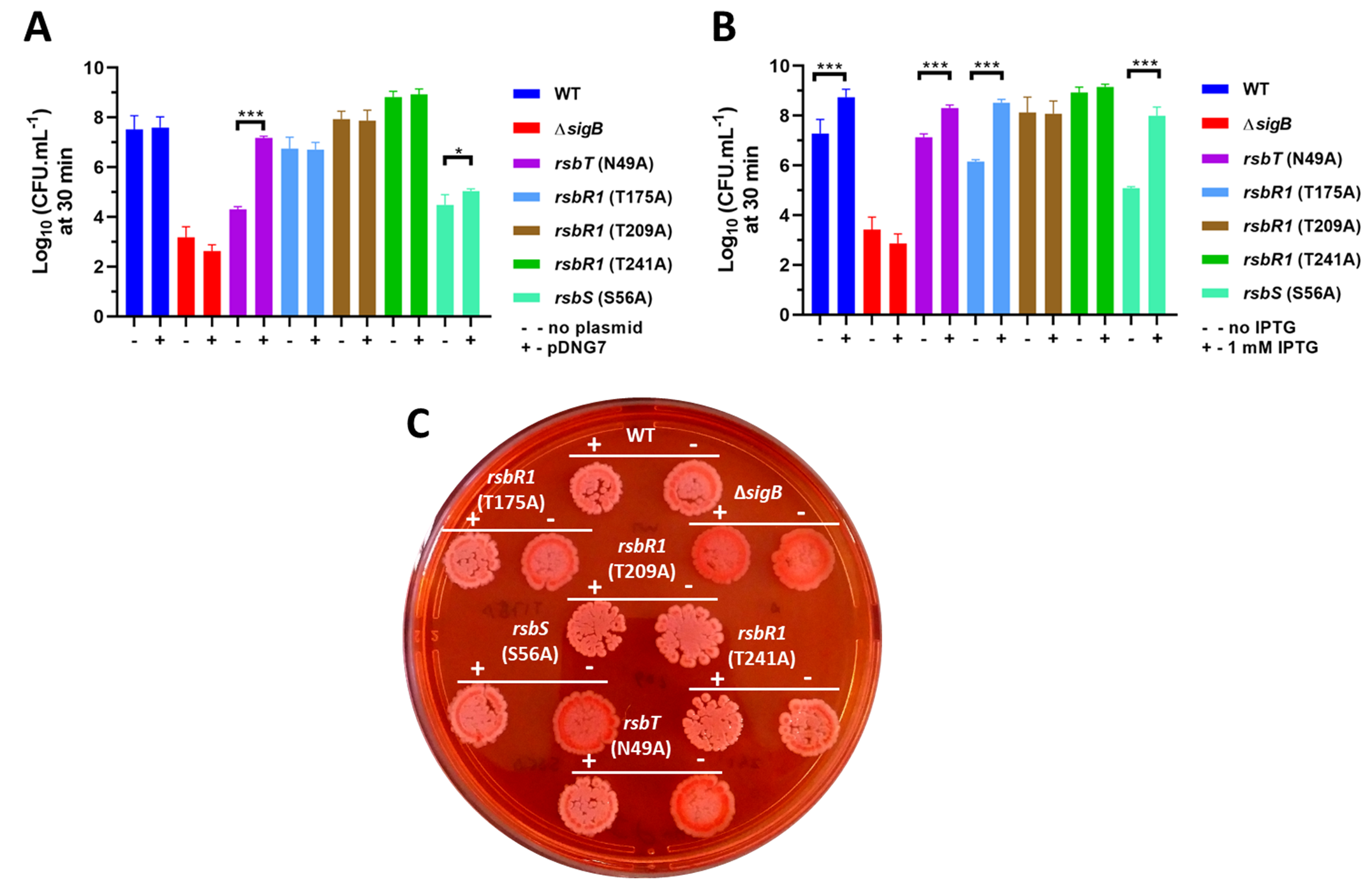

Supplement: S6 Fig — Mutant strains were genetically complemented with pDNG7, which contains the native rsbR rsbS rsbT open reading frame under the control of a leaky IPTG inducible promoter (Phelp::lacOid). (A) Complemented (+) and non-complemented (-) strains were grown to stationary phase without IPTG in BHI at 37°C and then challenged in BHI acidified to pH 2.5. (B) Complemented strains were grown with (+) and without IPTG (-) and subsequently challenged to pH 2.5. Results are expressed in Log10 (CFU.mL-1). (C) Stationary phase cultures were plated in BHI agar containing 25 μg.mL-1 of Congo Red and 1 mM of IPTG and further incubated at 37°C for 24 h and transferred to 30°C for more 3 days. (TIF) [file ppat.1010213.s006.tif]
